# Developing a New Computer-Aided Clinical Decision Support System for Prediction of Successful Postcardioversion Patients with Persistent Atrial Fibrillation

**DOI:** 10.1155/2015/527815

**Published:** 2015-05-18

**Authors:** Mark Sterling, David T. Huang, Behnaz Ghoraani

**Affiliations:** ^1^Biomedical Engineering Department, Rochester Institute of Technology, Rochester, NY 14623, USA; ^2^University of Rochester Medical Center, School of Medicine and Dentistry, Rochester, NY 14642, USA

## Abstract

We propose a new algorithm to predict the outcome of direct-current electric (DCE) cardioversion for atrial fibrillation (AF) patients. AF is the most common cardiac arrhythmia and DCE cardioversion is a noninvasive treatment to end AF and return the patient to sinus rhythm (SR). Unfortunately, there is a high risk of AF recurrence in persistent AF patients; hence clinically it is important to predict the DCE outcome in order to avoid the procedure's side effects. This study develops a feature extraction and classification framework to predict AF recurrence patients from the underlying structure of atrial activity (AA). A multiresolution signal decomposition technique, based on matching pursuit (MP), was used to project the AA over a dictionary of wavelets. Seven novel features were derived from the decompositions and were employed in a quadratic discrimination analysis classification to predict the success of post-DCE cardioversion in 40 patients with persistent AF. The proposed algorithm achieved 100% sensitivity and 95% specificity, indicating that the proposed computational approach captures detailed structural information about the underlying AA and could provide reliable information for effective management of AF.

## 1. Introduction

Atrial fibrillation (AF), the most common abnormal rhythm of the heart, is associated with significant morbidity and mortality and increases the risk of heart failure and stroke [1]. AF is the disorganized propagation of electrical activity in the atrium that prevents organized contractions. As a result, the atrial depolarization wavefront, the P-wave, measured during sinus rhythm (SR) devolves into a series of fibrillatory waves in the surface electrocardiogram (ECG). AF is known to be progressive in nature [2, 3]. The disease tends to worsen over time and the resistance to therapy increases. Paroxysmal AF is defined by self-terminating AF episodes that last no longer than seven days. Persistent AF is defined by AF episodes which lasts longer than seven days and typically requires medical intervention to be terminated. Lastly, if AF is sustained for over a year and all attempts to eliminate AF fail, the AF is defined as Permanent AF. Given the progressive nature of AF and potential risks of different AF therapies, it is critical to identify if a given therapy is effective. This could provide invaluable information for effective management of AF.

There are a variety of treatment options for AF, including both pharmacological and electrical cardioversion and also surgical methods. Direct-current electric (DCE) cardioversion is one noninvasive treatment for AF that applies controlled transthoracic electrical shocks synchronized to the R-wave of the patient [1] in order to end AF and return the patient to SR. The DCE cardioversion treatment may be either immediately unsuccessful or there may be a recurrence of AF in the following months, which means that AF cannot be terminated using the DCE cardioversion therapy. It was reported that this procedure is successful in around 80–100% of the patients; however, only 20–40% maintain SR within one year after the therapy [4]. Hence, a reliable test that could accurately predict the likelihood of SR maintenance after DCE cardioversion is important in order to weigh the benefits versus potential risks such as postshock bradycardia, malignant ventricular arrhythmias, and atrial thromboembolism [5]. Therefore, the objective of the present work is to develop a novel computational approach to analyze the electrocardiogram of AF patients before application of DCE cardioversion and predict the success of the therapy. Such a predictor could provide an important computer-aided clinical decision support system for therapy management of AF patients.

Over the past decade, several studies have attempted clinical and electrophysiological parameters to predict SR maintenance after DCE cardioversion of AF [6–11]. A central notion in AF therapy management is that irregularity of fibrillatory wave signals reflects the severity of the disease in an individual. Thus, several studies measured organization of atrial activity (AA) from the surface ECG as a measure of SR maintenance. Some of these algorithms include fibrillatory rate [6, 12, 13], harmonic decay [6], and entropy [14, 15]. However, none of the existing methods has been used in the routine clinical AF therapy management [16]. In the present study, we investigate AF organization beyond what has been performed in literature so far. Our method studies both the morphology and frequency of the fibrillatory waves during AF in an attempt to provide a strong and yet meaningful predictor for sinus rhythm maintenance after electric cardioversion. We apply a signal decomposition technique to examine the structure of AA at different decomposition levels for the purpose of prediction of the outcome of the DCE cardioversion in persistent AF.

A preprocessing technique is applied to extract the AA from the ECG. The matching pursuit (MP) technique [17] is used to decompose the AA signal into multiresolution time-frequency (TF) decompositions. The MP decomposition consists of a combination of wavelet atoms with two wavelet types (i.e., Coiflet1 and Symlet2) and 6 scales (*S*_0_ to *S*_5_). We investigate the type and scale of the wavelet types and scales that most accurately capture the structural changes relevant to SR maintenance and propose seven new MP features. Using a quadratic discriminant analysis (QDA) classification technique and leave-one-out cross validation, we evaluate the developed MP features on a database containing ECG from persistent patients who underwent DCE cardioversion. Details of the algorithm are outlined in Section 2; a validation of the features against clinical data and discussion is provided in Section 3. The paper is concluded in Section 4.

## 2. Methods

**Figure 1:**
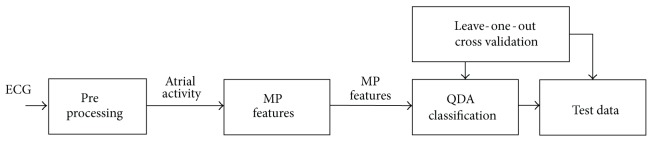
Overall outline of the study. Standard supervised learning approach is applied consisting of a feature extraction step followed by a classification step. Leave-one-out cross validation is used to evaluate the predictive power of our technique.

The proposed method consists of three stages: (i) preprocessing of the ECG signal, (ii) feature extraction, and (iii) classification and validation of the extracted features against clinical outcome.  Figure 1 depicts the overall outline of the proposed method.

### 2.1. ECG Database

The ECG data [18] was obtained from 40 persistent AF patients who had a successful external DCE cardioversion therapy. The study was approved by the local ethics committee of the enrolling organization and complied with the Declaration of Helsinki. Prior to cardioversion, a 10-minute 12-lead ECG (*f*_*s*_ = 1 kHz) was recorded for each patient. Twenty patients had maintained SR (AF-Free) after 2-week follow-up and 20 had a relapse of AF (AF-Relapse). The clinical characteristics of the patients, including medication and AF history, are given in Table 1. The proposed analysis was based on a single lead. Hence, we selected Lead V1, which has shown to provide the best atrial signal [19]. The proposed algorithm was applied on a 10-second long ECG signal, which was selected from the 10-minute ECG as explained in Section 2.2.

### 2.2. Preprocessing

The preprocessing stage is performed in three steps as follows.

*Noise and Baseline Wander Removal.* A bandpass filter with cutoff frequencies of 0.01 Hz and 50 Hz was used to remove the noise and baseline fluctuations in the ECG [20].

*Segment Selection.* Following the baseline removal step, the segments with a consistent QRST morphology are identified. In case of AF, it is common that the ECG contains more than one QRST morphology, which can increase the QRST residuals in the atrial activity extraction step and dilute the quality of AA for the further analysis. Hence in this step we identify the ECG segments that include steady QRST complexes. First, R-wave fiducial markers are placed at points of maximum absolute derivative on the QRST complexes. We construct a QRST template by averaging all of the QRST complexes in the ECG. We then compute the correlation between the QRST template and each beat and identify the segments with more than 90% correlation coefficient.

**Figure 2:**
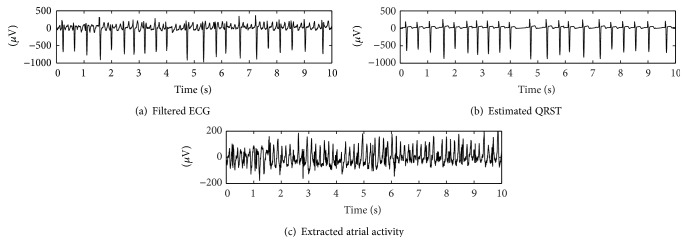
Illustrative example of an ECG waveform, the estimated QRST complex, and the extracted AA signal with kortusis values of 10, 13, and −0.45, respectively.

*Atrial Activity Extraction.* Several techniques have been used to cancel the QRST complexes and obtain the AA from the ECG [21, 22]. In this study, we employ the average beat subtraction method [23] which has been widely used in the literature. Using the QRST template that was computed in the previous step, at each fiducial marker, we fit the QRST template to the ECG and obtain the estimated QRST template from the ECG. Then, we subtract the estimated QRST template from the ECG to obtain the estimated AA signal. We evaluate the QRST removal by computing kurtosis as a measure of the AA estimation quality [24]. Finally, for each record we select a 10-second long excerpt with the lowest kurtosis.  Figure 2 illustrates the selected segment for one of the records.

### 2.3. Feature Extraction

MP decomposition is applied to the extracted AA signal and the MP features are extracted from the MP expansion coefficients.

*Matching Pursuit Decomposition*. MP is an iterative signal decomposition technique that expresses a signal *x*(*t*) as a linear combination of functions selected from an overcomplete dictionary of TF basis functions [17]. The algorithm has been successful in creating high-resolution TF representations of biomedical signals [25–27]. In this study, we apply the MP algorithm to the AA signal obtained from the preprocessing step. Consider

(1) $$\begin{array}{c} x\left(t\right)=\overset{M}{\underset{m=1}{\sum }}b_{m}A_{\left(W_{m},S_{m},T_{M}\right)}\left(t\right)+R_{x}^{M}. \end{array}$$

In (1), *x*(*t*) represents AA signal and *A*_(*W*_*m*_,*S*_*m*_,*T*_*M*_)_(*t*) is a wavelet with type, scale, and temporal location defined by *W*_*m*_, *S*_*m*_, and *T*_*m*_, respectively. *b*_*m*_ is the expansion coefficient for *A*_(*W*_*m*_,*S*_*m*_,*T*_*M*_)_(*t*), *M* is the number of iterations that are performed, and *R*_*x*_^*M*^ is the residue of *x*(*t*) after *M* iterations. In (1), the AA signal *x*(*t*) is projected onto an overcomplete dictionary of TF functions with a combination of different wavelet types and scales. At each iteration, the best correlated TF function is selected from the overcomplete dictionary by finding the maximum inner product of the current residue with each of the atoms in the dictionary (|〈*R*_*x*_^*M*^, *A*_(*W*_*m*_,*S*_*m*_,*T*_*M*_)_〉|). In the next iteration the residue is decomposed according to the same rules. After *M* iterations, the AA signal *x*(*t*) is expressed in the form of (1) where the first term on the right-hand side represents the decomposition of the original signal by the selected TF functions, and the second term is the residue at iteration *M*. For *M* large enough, it can be observed that the residue in (1) becomes negligibly small.

**Figure 3:**
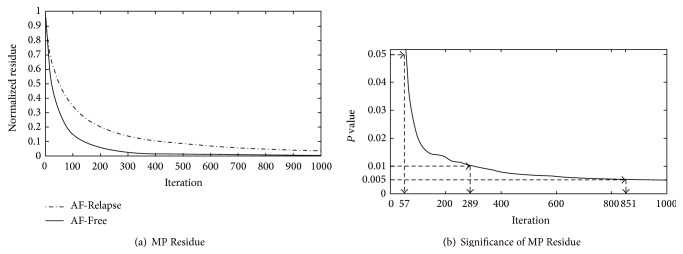
(a) Plots showing the behavior of the MP coefficients as a function of iteration number. This representation was used to find an appropriate number of iterations in our MP analysis. *M* was set equal to the average number of iterations required for the coefficients to reach less than 5% of their initial value. (b) The *P* values represent the statistical significance of the normalized expansion coefficient at each iteration. The vertical dashed lines mark the significance thresholds of 0.05, 0.01, and 0.005.

There are three ways of stopping the iterative process of MP. The iterations may proceed until the energy of the residue is less than a threshold, the value of the most recent expansion coefficient is less than a threshold, or the number of iterations reaches a preassigned maximum. In this study, we used a combination of the last two stopping methods and determined a fixed iteration number based on the average number of iterations required for the expansion coefficients to reach less than 5% of their initial value. Based on this analysis, we found that after *M* = 1,000 iterations, there is a negligible change in the expansion coefficients. Hence, we used *M* = 1,000 as the fixed stopping criterion. A plot of the expansion coefficients for an AF-Free and AF-Relapse example is shown in Figure 3(a).

**Figure 4:**
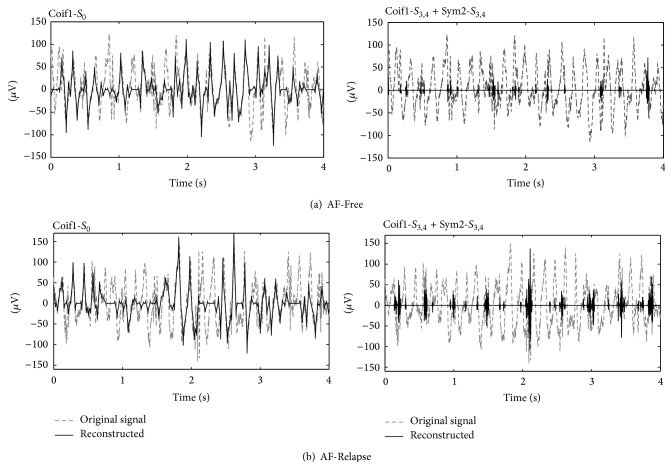
The reconstructed signals by MPF_Coif1,*S*_0__ and the combined reconstructed signals by MPF_Coif1,*S*_3__, MPF_Coif1,*S*_4__, MPF_Sym2,*S*_3__, and MPF_Sym2,*S*_4__ are displayed for an AF-Free (a) and AF-Relapse (b) case.

*MP Dictionary.* Two different wavelet types at six different scales (*S*_0_ to *S*_5_) are used in this study: Coiflet1 (Coif1) and Symlet2 (Sym2). We build a MP dictionary by pairing the two types of wavelets (i.e., *W*_1_ and *W*_2_). Then the MP decomposition projects each AA signal over the combined MP dictionary. We depict an example of AF-Free and AF-Relapse signal along with the signal decompositions in Figures 4(a) and 4(b), respectively. The plots on the left-hand side show the reconstructed signals by combining the components corresponding to Coif1 *S*_0_ and the right-hand side plots show the sum of the reconstructed signals related to MPF_Coif1,*S*_3__, MPF_Coif1,*S*_4__, MPF_Sym2,*S*_3__, and MPF_Sym2,*S*_4__.

*MP Features.* We performed the MP on each AA signal and obtained the decomposed wavelets and scales given by *A*_*W*_*m*_,*S*_*m*_,*T*_*m*__, *m* = 1,…, *M*. Thirteen MP features are extracted for each patient as explained in this section. However, only seven of these features contained a significant differentiation between the AF-Relapse and AF-Free data and were used in the final decision making algorithm.

The first MP feature was based on the expansion coefficient at *M* = 1,000 iterations. As can be seen in Figure 3(a), we realized that AF-Free cases present a faster decay rate compared to AF-Relapse cases. Such a behavior was expected as it can be hypothesized that the AF-Free cases present a more organized AA and are decomposed faster by the MP wavelets [28]. The AA signals from the AF-Relapse data contain more disorganized and incoherent structure and have a slower decay rate. Thus, we use the normalized expansion coefficient (i.e., MPF_Residue_ = *b*_*M*_/*b*_1_) at *M* = 1,000 iteration as the MP feature representing the decomposition decay rate. The logarithm of the normalized coefficient expansion is taken to further spread out the data points. We performed an exploratory statistical test to investigate if the expansion coefficient at a smaller number of iterations (i.e., *b*_*m*_/*b*_1_, where *m* < *M*) was a more appropriate choice for the quantification of AA organization. Using the Mann-Whitney *U* test, we calculated the *P* value of the normalized coefficient expansion for *m* = 1 to *m* = 1,000. As can be seen in Figure 3(b), the *P* value decreases as the iteration number increases. Any normalized expansion coefficient (*b*_*m*_/*b*_1_) with *m* > 851 can achieve a significant *P* value of <0.005.

**Figure 5:**
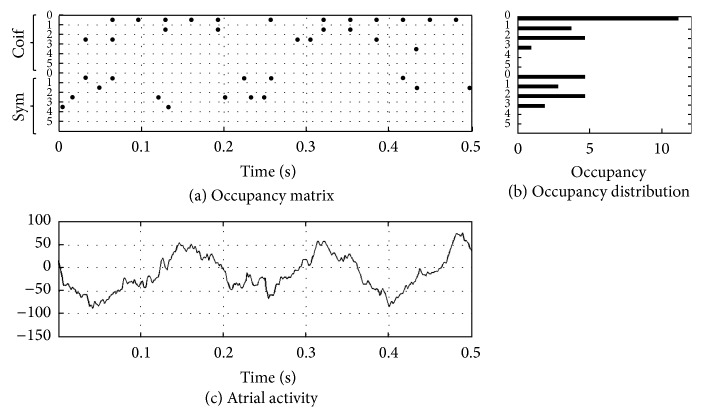
(a) The occupancy matrix is shown for an AF-Free case. Each black circle shows wherever there is a MP decomposition at a given time. The *y*-axis indicates the corresponding wavelet type and scale of each MP decomposition. (b) The occupancy distribution is shown for each wavelet type and scale. (c) The corresponding AA segments for the occupancy matrix. Only 0.5-second duration of the data is shown here for visualization purposes.

**Figure 6:**
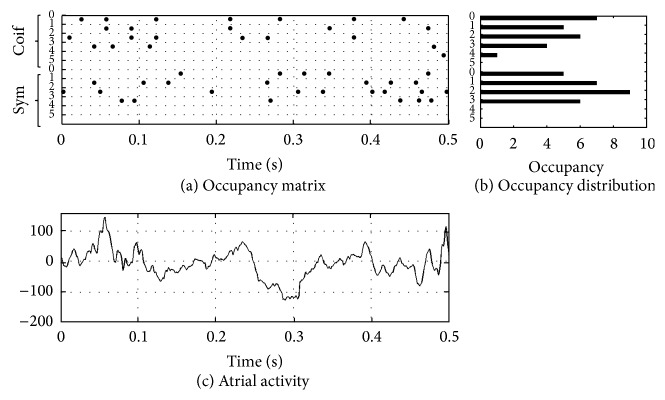
(a) The occupancy matrix is shown for a case of AF-Relapse. Each black circle shows wherever there is a MP decomposition at a given time. The *y*-axis indicates the corresponding wavelet type and scale of each MP decomposition. (b) The occupancy distribution is shown for each wavelet type and scale. (c) The corresponding AA segments for the occupancy matrix. Only 0.5-second duration of the data is shown here for visualization purposes.

The other twelve MP features are extracted based on the decomposition results as follows. We build two matrices for each wavelet type (*W*_1_ and *W*_2_) in a given dictionary: *O*_*W*_1__ and *O*_*W*_2__. These matrices which are called the* occupancy* matrices are constructed as follows:

(2) $$\begin{array}{c} O_{W_{1}}\left(i,j\right)=\left\{\begin{array}{cc} 1 & \text{if}\;\;W_{m}=W_{1}\\ 0 & \text{o}.\text{w}., \end{array}\right.\\ O_{W_{2}}\left(i,j\right)=\left\{\begin{array}{cc} 1 & \text{if}\;\;W_{m}=W_{2}\\ 0 & \text{o}.\text{w}., \end{array}\right.\\ \;\;\;\;\;\text{for}\;\;m=1,\ldots ,M, \end{array}$$

where *W*_*m*_ represents the wavelet type with scale and temporal location of *S*_*m*_ and *T*_*m*_, respectively, *i* = {0,…, 5} is the sacle value of *S*_*m*_, and *j* corresponds to the temporal location *T*_*m*_. A graphical representation of this process is shown in Figures 5 and 6 where two* occupancy* matrices of *O*_*W*_1__ and *O*_*W*_2__ are plotted for an example of AF-Free and an example of AF-Relapse, respectively. The plots display the analysis results of only 0.5 seconds of the AA data for visualization purposes. The first six rows show the probability of occupancy for Coif1 wavelets for scales *S*_0_ to *S*_5_, and the next six rows show this information for the Sym2 wavelets. In this plot, each black circle implies the presence of a decomposition at the given time and scale. Twelve features are extracted from each dictionary by summing over time as follows:

(3) $$\begin{array}{c} \text{MP}{\text{F}}_{W_{1,S_{i}}}=\underset{j}{\sum }O_{W_{1}}\left(i,j\right),\\ \text{MP}{\text{F}}_{W_{2,S_{i}}}=\underset{j}{\sum }O_{W_{2}}\left(i,j\right),\\ \;\;\;\;\text{for}\;\;i=0,\ldots ,5. \end{array}$$

In (3), we obtain the features as the total presence of a given wavelet type and scale in an AA signal. 

**Figure 7:**
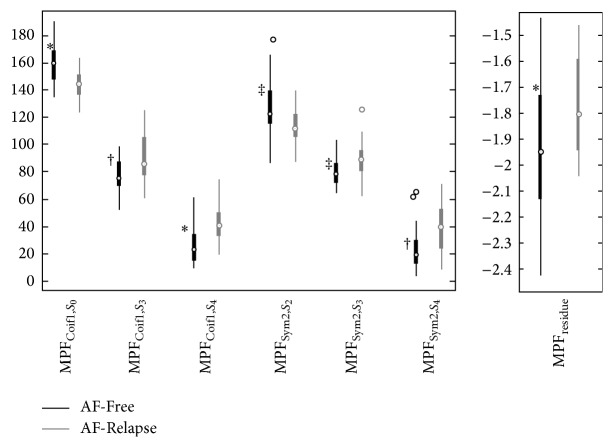
The seven statistically significant features are shown in this plot. ^*^ *P* value < 0.005, ^†^ *P* value < 0.01, and ^‡^ *P* value < 0.05.

*MP Feature Selection.* The MP features proposed in this study were evaluated using an exploratory statistical analysis. The purpose was to ensure that any subsequent learning technique we applied to the data would not be burdened by many irrelevant degrees of freedom. Thirteen MP features are extracted for each AA signal. We select the MP features that show a statistically significant correlation with the success of electric cardioversion. The statistical significance is determined for each MP feature using the Mann-Whitney *U* test, which is a nonparametric method for cases where the probability distribution of the data is not normal. This test is used in this study, because the MP features do not exhibit a Gaussian probability distribution. The Mann-Whitney *U* test results showed a statistical significance for only seven MP features. The values of these significant features are presented in Figure 7. The logarithm of the decay MP feature was taken to further spread out the data points. Because this feature had a different range than the other six features, it was shown in a separate plot. A total of seven MP features that are selected here are used in the classification stage: {MPF_Coif1,*S*_0__, MPF_Coif1,*S*_3__, MPF_Coif1,*S*_4__, MPF_Sym2,*S*_2__, MPF_Sym2,*S*_3__, MPF_Sym2,*S*_4__, MPF_Residue_}.

### 2.4. Classification

A label of “0” or “1” corresponding to the AF-Free and AF-Relapse cases, respectively, was attached to each of the feature vectors derived from all of the AA signals. The learning algorithm chosen for this study uses the quadratic discriminant analysis (QDA) which separates the AF-Relapse and AF-Free feature vectors by a quadratic surface. To evaluate the classification performance, we used a leave-one-out cross validation procedure where the data of one patient was withheld in each trial. Hence, the classification procedure is repeated in 40 trials corresponding to each of the patients and the training sets consist of the feature vectors from the entire database with the exception of the single patient withheld. The feature vectors from the patient under study are the test data. At every trial, the posterior probabilities of the left-out data corresponding to the AF-Free and AF-Relapse classes are recorded. A final receiver operating curve (ROC) is obtained using the collected posterior probabilities after all the 40 trials are completed.

## 3. Results

**Figure 8:**
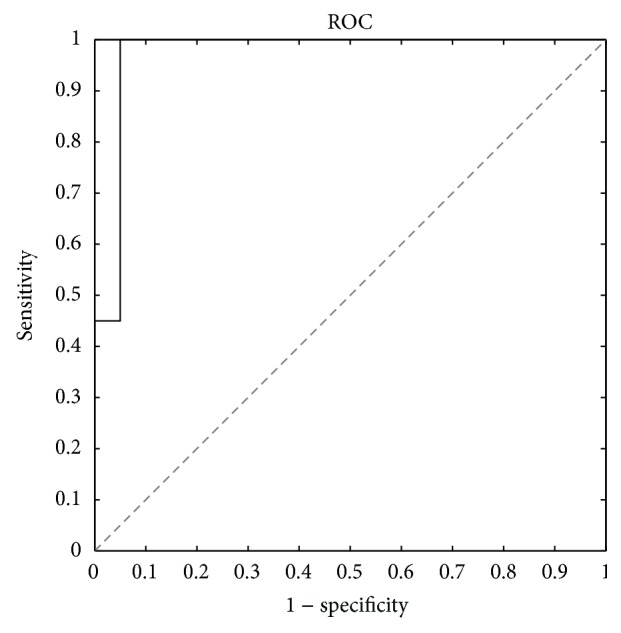
Receiver operating characteristic analysis of the QDA classification scores using leave-one-out cross validation. The AUC is 0.97 and the best sensitivity and specificity are 100% and 95%, respectively.

The proposed feature extraction and classification algorithm was applied to the dataset described in Section 2.1. The ROC of the QDA and leave-one-out cross validation is shown in Figure 8. According to this analysis, area under the curve (AUC) is 0.97, which is slightly higher than AUC of the linear discriminant analysis (LDA) method, which is 0.94. The best sensitivity and specificity values are 100% (20 out of 20) and 95% (19 out of 20), respectively. Except one case in the AF-Free class, all the data are perfectly classified using the novel features that are proposed in this paper.

### 3.1. Noise and QRST Residual

The preprocessing for the extraction of the AA signal is based on the average beat subtraction where the segments with a single morphology and the least QRST residuals are selected for the further analysis. It is common among AF patients that the ECG signal contains more than a single QRST morphology, which can result in extensive amount of QRST residue in the estimated AA signal. Another common problem is the changes in the QRST due to respiration and mismatches in the alignment of the QRST template with each QRST complex. In order to avoid computational artifact in the AA signal, we employ a correlation-based analysis to only consider the data with insignificant variability in the QRST complex. In our dataset, only 4 records consisted of a single morphology (i.e., no beat with the correlation of less than 90% with the QRST template). The remaining records had 32 ± 30 (8.5% ± 7.6%) beats that showed a correlation coefficient of less than 90% with the QRST template. In total, 4.6 ± 2.0 ECG segments (66 ± 42 seconds long) were selected for the AA extraction. Kurtosis was computed for each segment and the 10-second long ECG that showed the least kurtosis value was selected from each record. The average kurtosis value for the selected segments was −0.80 ± 1.01. We repeated the feature extraction and classification algorithm on randomly selected segments from each record (i.e., there was no constraint on the QRST correlation and kurtosis). The performance of the algorithm significantly dropped, which implies that selecting the noise and artifact-free segments is essential for the successful analysis of AA signals.

### 3.2. Relationship of Wavelet Type and Cardioversion Outcomes

Several observations can be made from the statistical analysis performed to select the significant MP features. Scale 0 (*S*_0_) of the Coiflet1 wavelet and Scale 2 (*S*_2_) of the Symlet2 wavelet model (i.e., decompose) the activations in the AF-Free signals. This behavior can be seen in Figure 7 which shows the elevated activities of MPF_Coif1,*S*_0__ and MPF_Sym2,*S*_2__ for the AF-Free data compared to the AF-Relapse data. It can also be observed from the right-hand side plots in Figure 4 that the AF-Relapse signal contains more elevated activations in the higher scales (i.e., *S*_3_ and *S*_4_) compared to the AF-Free signal. This can also be seen in Figure 7 where AF-Relapse data presents a higher activity at MPF_Coif1,*S*_3__, MPF_Coif1,*S*_4__, MPF_Sym2,*S*_3__, and MPF_Sym2,*S*_4__. In addition, comparing Figures 5 and 6 one can see that the occupancy matrix and distribution of the AF-Free signal are more concentrated at the lower scales while the AF-Relapse signal demonstrates a wider distribution. Hence, our observation is aligned with the literature [2, 35, 36] supporting that worsening AF is associated with a more disorganized atrial signal in the surface ECG. Our analysis suggests that the elevated activation of the higher scale wavelets in the AF-Relapse cases may be used as a predictor of disorganization and disturbances in AA signals.

### 3.3. Comparison with Other Related Studies for AF Progression

Table 2 lists results from a variety of previous studies proposed for the successful prediction of DCE cardioversion. It is worth mentioning that the results in this table were obtained using different datasets and the patient population used is important in explaining the differences among different studies. However, one may conclude that the proposed method provides a comparable if not better predictive capability compared to the other algorithms.

We assessed the performance of the atrial frequency rate (AFR) on our dataset as an important surface ECG statistic obtained during fibrillation. Previous studies, such as [13], show that AFR is correlated with endocardial measurements of cycle length. Moreover, as a measure of AA organization, AFR is significantly associated with risk of recurrence after therapy. Elevated AFR is generally understood to indicate a worsening of AF, perhaps associated with the progression of the disease via electrical remodeling [37]. We applied the AFR method to our dataset and found that the AFR was elevated in patients who had a recurrence of AF at follow-up (*P* = 0.012) and resulted in 58% and 75% sensitivity and specificity. Therefore, our analysis supported the significance of AFR in predicting the successful DCE cardioversion; however, as it is evident from these results we need to combine the AFR with some additional features in order to provide a successful aggregate score. For example, in Figure 4 the AFR did not find a significant difference between the AF-Free and AF-Relapse signals with the calculated AFR of 6.5 Hz and 6.4 Hz, respectively; however, the proposed multiresolution-based technique successfully differentiated the two cases. This result may be explained by the difference between the structures of the two methods. The proposed technique provides a distribution of the organization at different time and frequency scales while AFR is bounded by the time and frequency resolution of the Fourier Transform and can only provide the information about the overall frequency content over a given length of the signal. The performance of the proposed algorithm indicates that both the wavelet type and scale are important in predicting the successful postcardioversion patients. However, a larger population is required to further assess the success of the proposed MP-based analysis in a future study.

## 4. Conclusion

In this study, we proposed a novel analysis for the structure of the atrial activity to predict the success of DCE cardioversion AF therapy after 1 month following the therapy. We developed novel features from MP decomposition, performed a statistical evaluation, and selected 7 significant MP features. The extracted MP features were used in a quadratic discriminant analysis-based classification to predict the outcome of DCE cardioversion in our database. A leave-one-out evaluation demonstrated that our proposed algorithm provides a promising noninvasive indicator of the outcome with 100% and 95% sensitivity and specificity, respectively. Given the significant outcome, it may be concluded that our multiresolution-based signal decomposition technique yields novel insights into organization of the atrial activations that could improve the prediction of the successful postcardioversion patients. Further studies on wider databases could determine the reliability of the proposed computational approach as a new computer-aided clinical decision support system that could successfully predict the outcome of DCE cardioversion and may potentially guide the care of AF patients.

## Figures and Tables

**Table 1:** Clinical characteristics of the study population with respect to rhythm at follow-up.

Variable	AF-Free	AF-Relapse	*P* value
Age	68 ± 7	69 ± 7	N.S.
Male	16	17	N.S.
AF duration	128 ± 94	210 ± 94	N.S.
Other heart diseases			
Hypertension	6	7	N.S.
Ischemic heart disease	5	2	N.S.
Congestive heart failure	1	6	0.002
Valvular disease	1	3	0.04
Left atrial diameter	49 ± 6	51 ± 6	N.S.

Cardioactive drugs			

*β*-blocker	12	11	N.S.
Sotalol	3	3	N.S.
Class III antiarrhythmic agent	1	1	N.S.
Digitalis	2	7	0.05
Calcium channel blocker	3	5	N.S.

**Table 2:** Comparison of signal processing methods.

Method	Study size	Significance	Sensitivity	Specificity
P-wave duration 1997 [10]	35	0.001	73%	71%
Heart rate variability 2001 [29]	93	—	76%	90%
Fibrillatory rate 2003 [30]	44	0.021	—	—
Clustering of RR intervals 2004 [31]	66	0.034	—	—
P-wave duration 2005 [32]	118	0.0001	72%	77%
P-wave duration 2006 [8]	122493	0.02	90%	21%
Fibrillatory rate 2006 [13]	175	0.0001	79%	80%
Fibrillatory rate 2006 [6]	54	0.002	—	—
Harmonic decay 2006 [6]	54	0.0004	92%	47%
Sample entropy 2011 [14]	66	0.02	—	—
Wavelet transform 2007 [33]	30	—	100%	89%
P-wave dispersion 2011 [9]	26	0.001	86%	95%
Wavelet sample entropy 2008 [34]	40	—	95%	93%

Proposed MP-based method 2014	40	0.005	100%	95%
